# Development of an IgG4-RD Responder Index

**DOI:** 10.1155/2012/259408

**Published:** 2012-04-24

**Authors:** Mollie N. Carruthers, John H. Stone, Vikram Deshpande, Arezou Khosroshahi

**Affiliations:** ^1^Rheumatology Unit, Division of Rheumatology, Allergy, and Immunology, Department of Medicine, Massachusetts General Hospital, Boston, MA 02114, USA; ^2^Department of Pathology, Massachustts General Hospital, Harvard Medical School, Boston, MA 02114, USA

## Abstract

IgG4-related disease (IgG4-RD) is a multiorgan inflammatory disease in which diverse organ manifestations are linked by common histopathological and immunohistochemical features. Prospective studies of IgG4-RD patients are required to clarify the natural history, long-term prognosis, and treatment approaches in this recently recognized condition. Patients with IgG4-RD have different organ manifestations and are followed by multiple specialties. Divergent approaches to the assessment of patients can complicate the interpretation of studies, emphasizing the critical need for validated outcome measures, particularly assessments of disease activity and response to treatment. We developed a prototype IgG4-RD Responder Index (IgG4-RD RI) based on the approach used in the development of the Birmingham Vasculitis Activity Score for Wegener's granulomatosis (BVAS/WG). The IgG4-RD RI was refined by members of the International IgG4-RD Symposium Organizing Committee in a paper case exercise. The revised instrument was applied retrospectively to fifteen IgG4-RD patients at our institution. Those scores were compared to physician's global assessment scale for the same visits. This paper describes the philosophy and goals of the IgG4-RD RI, the steps in the development of this instrument to date, and future plans for validation of this instrument as an outcome measure.

## 1. Introduction

Measurement of disease activity is critical for longitudinal assessments in both observational studies and clinical trials. In the field of rheumatology, more than 250 assessment tools have been developed and validated to evaluate pathology, symptoms, function, and health status of patients with rheumatic diseases [1]. Such instruments should be compatible with regulatory requirements of the Food and Drug Administration (FDA) and generally require prospective studies for completion of the validation process [2].

IgG4-related disease (IgG4-RD) is an increasingly recognized immune-mediated disease that is characterized by a lymphoplasmacytic infiltrate enriched with IgG4-positive plasma cells and a distinctive storiform fibrosis of affected organs [3]. Commonly involved organs include the pancreas, biliary tree, orbits, salivary glands, and retroperitoneum, among many others. Organ involvement usually occurs in a metachronous but overlapping fashion. The serum IgG4 level is often but not always elevated [4]. Because of the novelty of IgG4-RD, little effort to date has been devoted to the development of outcome measures for this newly recognized condition.

A disease responder index is a tool designed to detect any changes in disease activity and identify improvement and worsening in the same and/or different organ systems. A responder index permits objective quantification of the treatment response by providing standardized outcome measures. Assessing clinical response and not simply serologic response is increasingly important to establish endpoints in randomized control trials.

No randomized control trials have been conducted for IgG4-RD treatment to date [5]. Management is based currently on small case series and observational studies. Glucocorticoids are the standard first-line treatment for IgG4-RD and patients whose disease has not reached an advanced stage of fibrosis generally respond well to this treatment, at least initially [5]. Recent data has shown that rituximab can be used successfully to treat IgG4-RD [6].

Two major features of IgG4-RD pose significant challenges for the development of outcome measures. The first is the complex, multiorgan system nature of this disease, which makes it difficult to summarize the state of disease activity across all organs. The second is the fact that the stage of disease activity can differ across organs, such that a patient can have active inflammation likely to respond to immunosuppression in one organ and advanced fibrosis (less likely to respond to treatment) in another.

We have developed an IgG4-RD responder index (IgG4-RD RI) for use as an outcome measure in an ongoing pilot trial of rituximab in this condition. We intend that this instrument will measure not only disease activity but will also incorporate features that capture the need for urgent treatment and catalogue disease-related damage. This paper is designed to provide information on the development and implementation of the IgG4-RD RI. We report the philosophy behind the development of the IgG4-RD RI to date, the steps taken to create the instrument through the enlistment of assistance of international experts in this condition, and the plans for completion of the IgG4-RD RI validation process.

## 2. Methods

### 2.1. Overview of the Instrument Development Approach

The IgG4-RD RI was designed to assess disease activity from visit to visit using clinician-generated assessments of both objective and subjective measures. The IgG4-RD RI uses a scoring system from 0–4 for each organ system or site and asks the clinician to rate the extent of disease activity and damage at the time of the clinical encounter. The IgG4-RD RI was revised by the Organizing Committee of the International IgG4-related Disease Symposium, held in Boston in October, 2011 [http://www2.massgeneral.org/pathology/symposium/IgG4_related_systemic_dis.asp]. This group was comprised of 39 experts from 9 countries, with subspecialty expertise in rheumatology, gastroenterology, allergy/immunology, nephrology, surgery, pathology, and radiology. Further revisions were made following a simulation exercise involving six paper case descriptions of real patients, completed by IgG4-RD symposium participants. Finally, both the IgG4-RD RI that emerged from these development steps and a physician global assessment (PGA) were used to assess the disease retrospectively in terms of disease activity and damage. The Pearson's correlation coefficient was then calculated to compare the IgG4-RD RI and PGA responses.

### 2.2. Model Disease Activity Tool

The IgG4-RD RI was modeled on the Birmingham Vasculitis Activity Score for Wegener's granulomatosis (BVAS/WG) [7]. The BVAS/WG is a formally validated and widely used instrument for the measurement of disease activity in granulomatosis with polyangiitis (formerly Wegener's granulomatosis) and microscopic polyangiitis, a pair of distinct but overlapping conditions often termed antineutrophil cytoplasmic antibody (ANCA)-associated vasculitides (AAVs) [8]. The BVAS/WG is a clinician-scored instrument in which each disease activity in each organ system is graded “persistent,” “worse,” or “none” at each clinic visit. The number of items of “persistent” or “worse” for each organ system is totaled and used to quantify the states of disease flare, persistent disease, or remission. The BVAS/WG was selected because of the experience of one of the authors (J. H. Stone) as a lead developer of this instrument; similarities between ANCA-associated vasculitis and IgG4-RD, including the propensities for multi-organ system involvement; the broad range of disease activity between flare and remission; the high frequency of disease-related damage (which must be distinguished from active disease); the absence of reliable biomarkers that necessitates reliance upon clinical indices for longitudinal assessments.

### 2.3. Scoring Sheet

**Figure 1:**
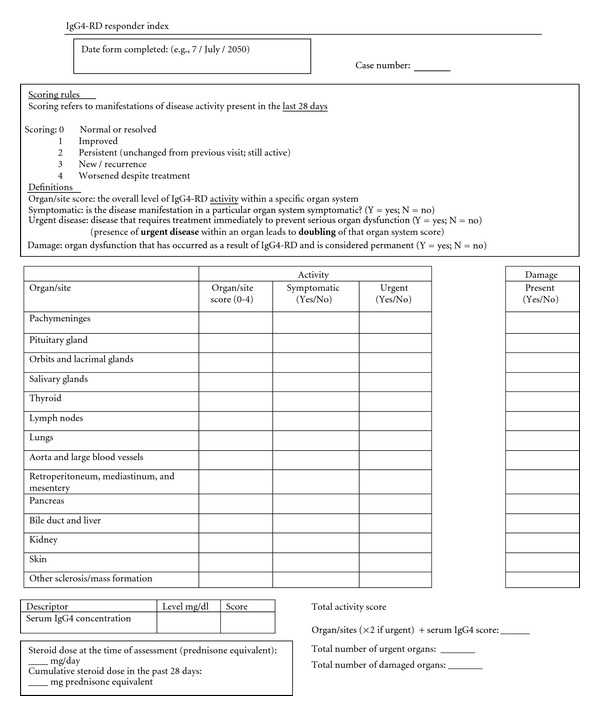
IgG4-related Disease Responder Index (IgG4-RD RI) Scoring Sheet this is a sample sheet of the IgG4-RD RI on which physicians score patient's disease activity at a given clinic visit.

The IgG4-RD RI, designed to emphasize ease of use, includes specific reminders to consider activity within all organs involved commonly in IgG4-RD (Figure 1). The scoring rules appear in the first box. At each visit, physicians enter a score from 0–4 for each organ/site affected, indicating whether the organ/site is normal, improved, new or recurrent, or worse on treatment. The physician also provides yes/no answers for each organ site to the questions of whether the disease is symptomatic; whether the disease activity requires treatment urgently; whether the organ dysfunction observed is related to damage rather than (or in addition to) active disease. At the end of this table, the serum IgG4 concentration in milligrams per deciliter is entered along with a score of 0–4, indicating whether the IgG4 concentration has improved, become newly or recurrently elevated, or increased despite treatment since the last visit. The scoring scheme for serum IgG4 concentration, therefore, parallels the schemes for individual organ system activity assessment. The cumulative glucocorticoid dose (in prednisone equivalents) since the last visit and total IgG4-RD RI score are then calculated.

### 2.4. Specific Interpretations of Individual Organ System Scores

The numbers for each organ score refer to disease activity, distinguished from organ dysfunction related to damage:

- “0” signifies the absence of active disease in that organ. A score of 0 is appropriate when the organ system has never been affected by active IgG4-RD, or when previously evident disease within that organ has resolved;
- “1” indicates that disease activity within an organ has improved but still persists to some degree;
- “2” indicates that the disease within that organ has remained persistent and unchanged since the last visit;
- “3” indicates the presence of new or recurrent disease activity;
- “4” refers to disease that has worsened despite treatment.

### 2.5. Organ Site

The organ sites were selected for inclusion in the IgG4-RD RI based on a review of the existing literature (Table 1) [3]. For ease of conceptualization, the sites of potential organ involvement are listed from head to toe. This structure is similar to that of the BVAS/WG scoring sheet, on which disease activity is scored by organ system, and each disease site is assigned a designation of normal, persistent disease activity, and new/worse disease activity, with numerical scores corresponding to each state [7]. Each organ site has specific disease manifestations common to IgG4-RD (the appendix). The IgG4-RD RI category of “other” organ/site involvement is important because the protean nature of this disease makes it impossible to capture all potential sites of disease. In addition, we anticipate that new clinical manifestations of this disease and possible even new sites of organ involvement will be described as the clinical phenotype of this disease is understood more fully.

### 2.6. Symptoms and Signs of IgG4-RD

Lists of the most common symptoms and signs within a given organ system are included in the IgG4-RD RI instructions (see the appendix), principally as a reminder to the clinician of the possible disease manifestations to consider when scoring disease activity and damage. The physician simply denotes on the form the presence or absence of symptoms for a given IgG4-RD site. This feature of the instrument ensures that a subjective measure is included. Good clinical judgment and a thorough knowledge of the disease manifestations of this condition are essential, as with any clinical responder index.

### 2.7. Urgency

Capturing the need for urgent treatment is an important aspect of the IgG4-RD RI. Some disease manifestations of IgG4-RD require the immediate institution of treatment to prevent permanent organ damage. For example, IgG4-related sclerosing cholangitis can lead to cirrhosis within several months of diagnosis and requires the prompt initiation of therapy. In contrast, the lymphadenopathy of IgG4-RD remains unchanged for prolonged periods in many patients and may never require treatment. The “urgent” column in the IgG4-RD RI is designed to capture aspects of the disease that require the immediate start of immunosuppression in order to preserve organ function.

The score for an organ site is doubled when the need to initiate treatment for active IgG4-RD at a particular or organ/site is considered urgent. For example, if a patient has new IgG4-related sclerosing cholangitis, the total score for that organ/site would be 6 instead of 3. Similarly, if the patient's biliary status has worsened despite therapy since the time of the last visit and an urgent escalation of therapy is required to treat the IgG4-related sclerosing cholangitis, then that organ score would be 8 rather than 4. Only the score of the individual organ site is doubled in this setting, not the total IgG4-RD RI score.

### 2.8. Damage

The concept of damage is related directly to that of disease activity. Consequently, the two concepts must be considered in tandem. Organ damage results from active disease and in some cases both active disease and damage can be present in the same organ system simultaneously. In other cases, organ dysfunction is related more to damage than to active IgG4-RD. Immunosuppression must be targeted to active IgG4-RD, not to damage resulting from previously active therapy. The most appropriate use of immunosuppression is to control active disease and prevent disease-related damage. It is particularly ideal to employ immunosuppression at a stage of disease when the histopathology is characterized by a lymphoplasmacytic infiltrate rather than a predominance of acellular fibrosis [9].

The clinical assessment of damage can be challenging. Radiographic studies such as computed tomography (CT) and positron emission tomography with CT (PET-CT) can aid the clinician in determining which organs have been damaged. For example, even conceding that active disease might be present simultaneously with damage within the pancreas, the finding of atrophic changes by CT scan within that organ would be considered to be the result of damage. In such a case, both active disease and disease-related damage should be scored.

### 2.9. Serum IgG4 Concentration

Scoring also includes a consideration of the serum concentration of IgG4. The serum IgG4 level may become elevated in a patient experiencing an active flare [6, 10]. However, not every patient with IgG4-RD has an elevated serum IgG4 level at baseline, even before treatment. It is well established that classic IgG4-RD can be active in the absence of elevated serum IgG4 concentrations. The IgG4-RD serum level in the IgG4-RD RI is scored according to normal, improved, persistent, new, recurrent, or worsened despite treatment. Patients with rising serum IgG4 levels would have higher scores indicating worsening disease activity.

### 2.10. Total Scoring

The sum of the disease activity in all of the organ sites plus the serum IgG4 concentration score (also graded on a 0–4 scale) yields the total activity score. An individual active organ site is doubled for urgency and added to the other organ sites. This number can be compared between visits to assess the disease activity over time as well as being used for a clinical trial endpoint. The longitudinal recording of damage, though not included in the overall disease activity score, is essential to the formulation of the patient's overall outcome.

### 2.11. Glucocorticoid Use

Glucocorticoids are the cornerstone of IgG4-RD treatment, and most patients respond promptly to this treatment, at least initially. Thus, careful recording of the dose of prednisone (or prednisone equivalent) in the interval between the current visit and the preceding one is essential to a full understanding of the degree of disease activity.

## 3. Results

### 3.1. Simulation Exercises

**Figure 2:**
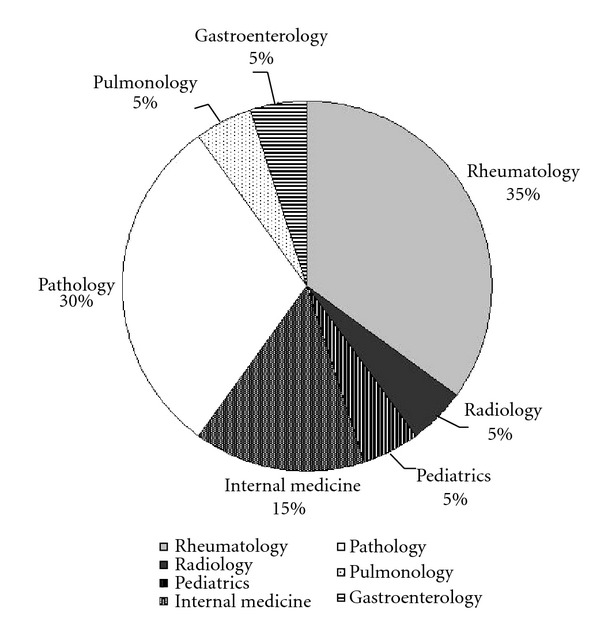
Subspecialties of IgG4-RD RI Simulation Exercise Participants (*n* = 21) there were 21 participants completing the simulation exercises and the pie chart represents the percentage breakdown for each medical subspecialty.

The IgG4-RD RI went through several development stages and iterations before arriving at its current format. A simulation exercise using paper case descriptions of six real patients was sent to attendees of the *International IgG4-RD Symposium* (held in Boston, MA, USA October 2011). Participants were asked to score the simulation exercises using the IgG4-RD RI. The participants received written instructions on how to apply the IgG4-RD RI but did not attend a training session (the appendix). Twenty-one individuals participated in this exercise, providing valuable feedback from a cross-section of investigators interested in IgG4-RD. The physicians who completed the exercises included a variety of subspecialists, particularly rheumatologists and pathologists (Figure 2). 

The simulation exercises were presented as clinical vignettes, including data from histories, physical examinations, laboratory results, and radiologic findings for each case. Accompanying each clinical vignette was at least one clinical photograph, radiology study, or histologic image to illustrate the case effectively. The physicians then used all of the information presented in the simulation exercise to complete a separate IgG4-RD RI scoring sheet for each case. The results were scored for each participant against standardized answers prepared by consensus of the four authors.

The purpose of this exercise was to solicit feedback on the IgG4-RD RI from physicians who were experts in the evaluation of patients with this disorder from different perspectives. Several common scoring errors were observed in this exercise. These included scoring organ involvement in which clinical symptoms and signs had resolved entirely as improved but persistent (i.e., “1”) rather than resolved (i.e., “0”). Another scoring discrepancy resulted from incorrectly scoring patients off treatment who were recurrent (“3”) as if they were receiving treatment (“4”). A third error was failing to double the organ/site score, when disease requiring treatment urgently was present. Comments from the participants in this exercise contributed substantially to important revisions of the draft instrument. We reformatted the scoring sheet in order to address the common scoring differences from the simulation case exercises.

### 3.2. Retrospective Application of the IgG4-RD RI

**Figure 3:**
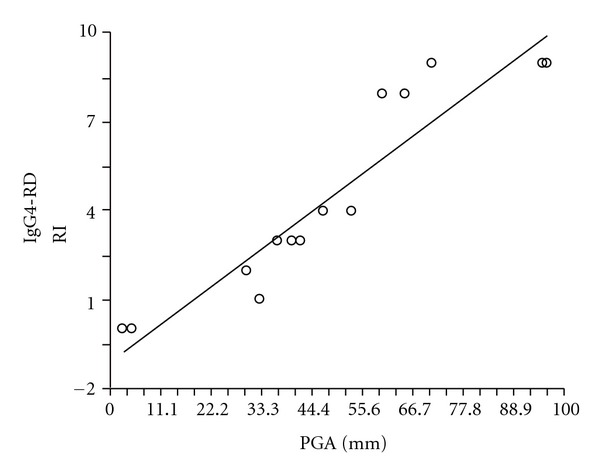
Correlation between physician global assessment (PGA) and IgG4-RD RI the PGA and IgG4-RD RI were compared using linear regression.

The next step in the development of the IgG4-RD was the retrospective use of the instrument for fifteen individual clinic and in-patient evaluations among patients in the Massachusetts General Hospital IgG4-RD Registry. Two blinded rheumatology experts scored an IgG4-RD patient visit using either the IgG4-RD RI or the physician's global assessment scale (PGA). These results were then compared (Figure 3). The Pearson's correlation coefficient was 0.93 (*P* < 0.0001).

## 4. Discussion

As the field of IgG4-RD is poised to move beyond the descriptive phase of the disease, validated outcome measures are required to advance the understanding of this condition and the assessment of new treatment approaches. The current iteration of the IgG4-RD RI marks an important step toward the availability of useful outcome measures in this disease. We anticipate that additional validation steps for this instrument will be required, but this paper describes accurately the philosophy and goals behind the IgG4-RD RI. Its methods and appendices will serve as important guidance documents in the future.

The development efforts to date have created a one-page instrument supported by the instruction manual shown in the appendix. Data included on this single page include indications of disease activity across a full spectrum of potential organ involvement; the serum IgG4 concentration; assessments of the need for treatment on an urgent basis; the recording of damage in organ systems; the sum of recent glucocorticoid use. Expertise with the use of the IgG4-RD RI may, therefore, become a concise and important tool for clinical trials and other investigations related to this disorder.

Although the developers of the IgG4-RD RI have relied significantly upon the BVAS/WG in creating this instrument, the IgG4-RD RI differs in important ways from the BVAS/WG. The “urgent” column in the IgG4-RD RI highlights features of the disease that require the prompt institution of treatment and is, therefore, analogous to the “major” designations given to some organ system manifestations in the BVAS/WG [7]. However, the BVAS/WG does not record disease damage on the same page. Rather, clinical trials in AAV have generally used a separate instrument, the Vasculitis Damage Index [11], for this purpose. Although it is critical that the concepts of disease activity and damage be kept separate and recorded appropriately during clinical assessments, it may be useful to have an indication of damage on the same one-page case report form even if damage does not contribute to the overall disease activity score. This model matches more closely the decision-making process that clinicians undertake on a daily basis in encounters with patients: are the signs of organ dysfunction due to active disease, or are they more accurately a reflection of damage rather than a process that requires more intensive immunosuppression?

The IgG4-RD RI will find its greatest use in the research setting, either in the context of clinical trials or in other types of investigations that require the careful longitudinal assessments of patients' clinical status. Because consistency of its application from visit to visit is critical, it will be most useful to ensure whenever possible that the same investigators complete the IgG4-RD RI for the same patient across all visits.

Significant debate now exists within the community of IgG4-RD investigators about the utility of serum IgG4 concentration measurements in the diagnosis and management of this disorder [12]. Inclusion of the serum IgG4 concentration in the IgG4-RD RI at this point permits an analysis of the value of this measurement in the context of other organ disease assessments. We hypothesize that further analysis of these data will confirm the utility of serial measurements, at least in a subset of patients. This hypothesis, however, requires confirmation through studies of larger numbers of patients in a variety of states of disease activity.

The simulation case exercises illustrated some shortcomings in early iterations of the IgG4-RD RI that led to appropriate revisions of the original index. The experience with the simulation exercise highlighted the importance of adequate training with the instrument prior to its use in the research setting. The IgG4-RD RI is simpler than many clinical assessment tools for multiorgan diseases, but both a thorough understanding of the clinical breadth of IgG4-RD itself and a high degree of familiarity with the index are required in order to employ it effectively. We anticipate that a focused period of instruction for investigators in the context of a formal training course will be required before this tool can be used in the context of a clinical trial.

In conclusion, progress in IgG4-RD will be contingent upon the ability to assess patients rigorously in a longitudinal manner, using validated outcome measures. The IgG4-RD RI described in this paper represents a broad effort at the development of a disease activity and responder index that can be employed in clinical trials and other investigations of patients with this emerging immune-mediated condition. The next steps in validation will include a multicenter study of patients recruited from a core group of sites with extensive experience in the diagnosis and management of this disease.

## Supplementary Material

The supplementary appendix includes the instructions for use of the IgG4-related disease responder index (IgG4-RD RI). This instruction manual describes how both disease activity and damage are recorded on the IgG4-RD RI scoring sheet (Figure 1). Scoring rules are described for the organ/site scores as well as serum IgG4 concentration and total scores. The definition of a damaged organ is provided and how to record damage. Finally, a version of the IgG4-RD RI that includes all of the organ/site common disease manifestions is included.

## Figures and Tables

**Table 1:** Diseases commonly associated with IgG4-related disease.

IgG4-related spectrum diseases
Idiopathic hypertrophic pachymeningitis
Orbital pseudotumor
Mikulicz's disease
Kuttner's tumor
Eosinophilic angiocentric fibrosis
Riedel's thyroiditis
Idiopathic cervical fibrosis
Pulmonary inflammatory pseudotumor
Chronic sclerosing aortitis
Inflammatory abdominal aortitis
Autoimmune pancreatitis
Sclerosing cholangitis
Retroperitoneal fibrosis
Inflammatory pseudotumor of the kidney

## Appendix

For more information please refer to IgG4-RD RI Instructions Manual. (See Supplementary Material available online at doi:10.1155/2012/259408.)
