# Supplementary material for: Development of an IgG4-RD Responder Index
Source: Int J Rheumatol. 2012 Apr 24;2012:259408. doi: 10.1155/2012/259408 (PMC3348627; doi:10.1155/2012/259408)
Supplement: Supplementary file 1 — The supplementary appendix includes the instructions for use of the IgG4-related disease responder index (IgG4-RD RI). This instruction manual describes how both disease activity and damage are recorded on the IgG4-RD RI scoring sheet (Figure 1). Scoring rules are described for the organ/site scores as well as serum IgG4 concentration and total scores. The definition of a damaged organ is provided and how to record damage. Finally, a version of the IgG4-RD RI that includes all of the organ/site common disease manifestions is included. [file 259408.f1.pdf]

Instructions Manual

IgG4-Related Disease Responder Index (IgG4-RD RI)

Approach to Assessing Disease Activity, Damage, and Treatment Response

The IgG4-RD Responder Index (IgG4-RD RI) is completed at each study visit.

Both activity and damage are recorded on the IgG4-RD RI, but they are recorded in separate parts of the index.

Disease activity refers to ongoing manifestations of IgG4-related inflammation within an organ. In contrast, damage refers to organ system dysfunction that has occurred as a result of active IgG4-RD, whether or not the disease is still active in that organ system.

Each assessment relates to the level of IgG4-RD activity on the day the patient was evaluated and to the 28 days preceding that assessment.

Clinical symptoms and signs that are associated with damage as opposed to active disease are recorded as damage within the particular organ system, not as activity.

Patients may have both damage and activity in the same organ at the same time. In that even, both damage and activity should be recorded on the IgG4-RD RI.

Standard Tests at Periodic Intervals

All patients will undergo a specified set of tests according to their organ involvement and discretion of the clinician at entry and at six months. These tests may include :

- Computed tomography of the chest and abdomen
- Additional imaging of other organ involvement, as appropriate
- Complete blood count with differential
- Serum chemistry profile, to include blood urea nitrogen, creatinine, and tests of liver function: bilirubin, alkaline phosphatase, and alanine and aspartate aminotransferase
- Urinalysis with microscopic examination of the urine sediment

These tests may be repeated at other times as appropriate to clinical care. A serum IgG4 concentration will be measured at each visit, as this assay is part of the IgG4-RD RI.

Recording of Activity

Three columns in the IgG4-RD RI describe aspects of disease activity: the "Organ/Site score" column, the "Symptomatic" column, and the "Urgent" column. We define these columns below.

Organ/Site score: The overall level of IgG4-RD activity within a specific organ system. Each organ or site is given a separate score, and these scores from a total of 15 organs & sites (including the serum IgG4 concentration) are summed to give the total IgG4-RD Activity Score.

Here is the scoring system for each overall organ or site:

0 – If the organ/site is normal, or if previous IgG4-RD disease activity has resolved
1 – If the disease activity in the organ/site has improved since the previous evaluation
2 – If the disease activity is persistent (Unchanged from previous visit; still active)
3 – If there is new disease activity at an organ or site where there was previously none or recurrence of the disease activity in an organ which was symptom free in the previous visit(s).
4 – If the disease activity is worse in that organ/site compared to the previous evaluation, *despite treatment*.

Individual disease manifestations listed under each organ system are intended simply as a reminder to the clinician to consider these potential disease features. They are not scored separately if present but rather are reflected in the overall organ/site score.

Symptomatic: Is the disease manifestation in a particular organ system symptomatic? (Y = yes; N = no)
If yes is the answer, the symptom needs to be specified.

Not all features of active IgG4-RD are symptomatic. Examples of this include lymphadenopathy in some cases; pulmonary nodules in others; and proteinuria in others.

In some organ systems in some patients, active disease may not require therapy. As an example, not all cases of IgG4-related lymphadenopathy need to be treated immediately (and some may never require treatment). Conversely,

disease that is asymptomatic may be sufficiently important or dangerous as to require prompt treatment. Aortitis is an example of this.

Urgent disease: Does the disease in this organ/site require treatment immediately to prevent serious organ dysfunction (Y = yes; N = no)

Some features of IgG4-RD require treatment urgently to prevent serious organ dysfunction. Examples include biliary tract disease, aortitis, and pachymeningitis. If an IgG4-RD feature is sufficiently severe as to require immediate treatment to prevent serious organ dysfunction, that organ/site disease is considered to be urgent. Urgent disease at an organ or site is weighted times two.

Urgent disease does not necessarily reflect disease extent, i.e., the number of organs involved by a disease. In some patients, IgG4-RD may be widespread and involve multiple organ systems or sites, yet not be worrisome enough in any body area to require treatment immediately. In contrast, disease that is apparently isolated to one organ system (e.g., the liver/biliary tract) may be severe enough to require treatment urgently.

Recording of Damage

One column in the IgG4-RD RI relates to damage as opposed to activity. This is the “Damage” column, separated on the right from the activity columns on the left.

Damage: Organ dysfunction that has occurred as a result of IgG4-RD and is considered permanent (Y = yes; N = no)

“Damage” refers to organ system dysfunction that has occurred as a result of active IgG4-RD, whether or not the disease is still active in that organ system. Damage is regarded as a permanent organ/site “scar” that will not improve, even with appropriate treatment for IgG4-RD.

IgG4-RD Disease Responder Index

Date form completed: (e.g., 7 / July / 2050)
__/___/ ____

Case number: ____________

Scoring Rules:

This case report form refers to manifestations of disease activity present in the <u>last 28 days</u>

Scoring: 0 Normal or resolved
1 Improved
2 Persistent (Unchanged from previous visit; still active)
3 New / Recurrence
4 Worsened despite treatment

Definitions:

Organ/Site score: The overall level of IgG4-RD <u>activity</u> within a specific organ system

Symptomatic: Is the disease manifestation in a particular organ system symptomatic? (Y = yes; N = no). if yes, specify the symptom.

Urgent disease: Disease that requires treatment immediately to prevent serious organ dysfunction (Y = yes; N = no) (*The presence of urgent disease within an organ leads to* ***<u>doubling</u>*** *of that organ system score*)

Damage: Organ dysfunction that has occurred as a result of IgG4-RD and is considered permanent (Y = yes; N = no)

	Activity			Damage
Organ/Site	Organ/Site Score (0-4)	Symptomatic (Yes/No)	Urgent (Yes/No)	Present (Yes/No)
Pachymeninges<br>• headache<br>• cranial nerve palsies<br>• radiculomyelopathy<br>• meningeal enhancement<br>• Other (specify):				
Pituitary gland<br>• visual field abnormalities<br>• endocrine dysfunction<br>• pituitary mass<br>• Other (specify):				
Orbits and lacrimal glands<br>• proptosis<br>• lacrimal gland				

swelling<br>• retro-bulbar mass<br>• Other (specify):				
	**Activity**			**Damage**
Organ/Site	Organ/Site Score (0-4)	Symptomatic (Yes/No)	Urgent (Yes/No)	Present (Yes/No)
Salivary glands<br>• swelling<br>• sicca symptoms<br>• Other (specify):				
Thyroid<br>• Nodular enlargement<br>• Diffuse enlargement<br>• Other (specify):				
Lymph nodes<br>• lymphadenopathy<br>• Other (specify):				
Lungs<br>• pulmonary nodules or mass<br>• interstitial pneumonia<br>• pulmonary fibrosis<br>• pleural effusion and thickening<br>• Other (specify):				
Aorta / large blood vessels<br>• aortic aneurysm<br>• aortic dissection<br>• wall thickening and inflammation<br>• Other (specify):				
Retroperitoneum, Mediastinum, and Mesentery<br>• mass lesion<br>• ureteral stenosis<br>• superior vena cava syndrome<br>• Other (specify):				
Pancreas<br>• obstructive jaundice<br>• pancreatitis<br>• pancreatic mass<br>• diffuse pancreatic				

enlargement<br>• Other (specify):					
	Activity				Damage
Organ/Site	Organ/Site Score (0-4)	Symptomatic (Yes/No)	Urgent (Yes/No)		Present (Yes/No)
Bile duct and liver<br>• abnormal liver function tests<br>• hepatic mass<br>• Other (specify):					
Kidney<br>• sterile pyuria<br>• hematuria<br>• subnephrotic proteinuria<br>• mass lesion<br>• Other (specify):					
Skin<br>• nodular lesions<br>• Other (specify):					
Other<br>Sclerosis/mass formation in<br>• breast<br>• prostate<br>• maxillary sinus<br>• nasal septum<br>• pericardium<br>• Specify other organ(s) involved:					

Score	Level mg/dl	Descriptor
		Serum IgG4 concentration

Total Activity Score (Organ/Site plus serum IgG4 score): _______

Total number of Urgent organs involved: _______

Total number of Damaged organs: _______

Steroid dose at the time of assessment (prednisone equivalent): ________ mg/day

Cumulative steroid dose in the past 28 days: ________ mg prednisone equivalent
